# Social networks as a protective factor for worsened self-perceived health status related to self-perceived changes in loneliness and health conditions in adults aged 50+ during the COVID-19 outbreak

**DOI:** 10.1016/j.heliyon.2023.e20529

**Published:** 2023-09-29

**Authors:** Shay Musbat, Inbal Reuveni, Racheli Magnezi

**Affiliations:** a Department of Management, Health Systems Management Program, Bar-Ilan University, Ramat Gan 5290002, Israel; b Department of Psychiatry, Hadassah Hebrew University Medical Center, Ein Kerem, Jerusalem 9112001, Israel

**Keywords:** Aging, COVID-19, Electronic communication, Europe, Loneliness, Self-perceived health, Social networks

## Abstract

The coronavirus disease 2019 (COVID-19) has emerged as a global pandemic, leading millions of people to change their lifestyles, especially older individuals who are the most at-risk population. Social isolation, the main preventive action to slow the pandemic's spread, reduced and drastically limited social connections, increasing older individuals' loneliness and stress, and worsening their health. We examined the connection between self-perceived changes in loneliness, the existence and type of social contact (face-to-face/electronic), and health conditions on self-perceived changes in health status during the outbreak, analyzing 51,778 individuals aged 50 plus from the Survey of Health, Ageing and Retirement in Europe (SHARE) database Wave 8 beta (June–August 2020). We found that the odds for worsened self-perceived health status were 249% higher among individuals who reported increased loneliness compared to the non-increase group and were lower in individuals with face-to-face contact (31%) or electronic contact (54%) during the outbreak. In addition, the odds for worsened self-perceived health status were higher for individuals with hypertension (17%), cancer (19%), chronic lung disease (25%), heart problems (27%), and other illnesses (32%). Based on the results obtained, electronic contact has shown a stronger connection as a protective factor for worsened self-perceived health since the outbreak compared to face-to-face interactions. Thus, adopting a policy that encourages the usage of electronic communications could reduce the burden on the healthcare system, particularly during pandemics, while improving patient health outcomes and minimizing pandemic-related health risks. This approach is especially important for older individuals, for whom any departure from home can cause an additional risk of exposure to the virus.

## Highlights

• Increased loneliness is connected to worsening self-perceived health in the outbreak.
• Electronic contact buffers the aggravation of self-perceived health in older adults.
• Electronic contact: stronger connection to better perceived health than face-to-face.

## 1 Introduction

The coronavirus disease 2019 (COVID-19) is a respiratory infection caused by the virus severe acute respiratory syndrome coronavirus 2 (SARS-CoV-2) [1]. COVID-19 causes several mild flu-like symptoms like fever, cough, fatigue, and in some cases, gastrointestinal infection symptoms [2]. Few patients with complications developed acute respiratory distress syndrome, acute cardiac injury, secondary infection, and several died [3]. The COVID-19 outbreak has emerged as a global pandemic and become an international public health major concern [4], leading millions of people globally to change their lifestyles. Close social connections, personal outdoor leisure activities, professional occupations, and educational activities were altered to slow the transmission of the virus, as social distancing became the main preventive action, essential for controlling the virus spread [5]. As the new reality took over and altered life routines, older individuals who were among the most at-risk populations [6], stayed in their homes and avoided physical contact with the outside world. The decrease in social relationships increased feelings of social isolation and loneliness [5,7,8].

Loneliness is commonly defined as an unsettling subjective feeling of being alone that accompanies the perception that the quality of one's existing social relationships does not satisfy his/her social needs, and social isolation describes an objective state of individuals' amount and frequency of social interaction patterns [5,9,10]. As for social isolation, it is viewed as an objective absence or paucity of social interactions [5,10]. Loneliness has been linked with an increased risk of cardiovascular disease [11], hypertension [12], and heightened inflammatory responses to stress [13]. Social isolation was found to be an independent risk factor for high levels of inflammatory markers shown to predict coronary heart disease and death from coronary heart disease in adults aged 40–75, even in those without a history of heart attacks [14]. In addition, social isolation and loneliness have shown a connection with increased mortality in the older population [15]. Loneliness and social isolation impact health by engaging behavioral and psychological mechanisms, as well as pathophysiological pathways such as increased cortisol levels, which is the primary human stress hormone [16].

Social relationships can be described in two different terms — those of, social support and social networks. Though describing different aspects of social relationships, they both can be viewed as important contributors to the individual's health [17]. Social support is defined as having interpersonal relationships and supportive individuals [18] and is represented by such types as emotional, instrumental, and informational support [19]. As for social networks, these are the individual's web of relationships, including family, friends, neighbors, and other connections in the social environment [18]. Social networks are the foundation that enables the provision of social support [20]. While recognizing the role played by social support in protecting individuals from psychosocial and physical impairments, the present paper, among other phenomena, focuses on social networks — face-to-face or electronic — and their correlation with self-perceived changes in health status.

In order to explain the relationship between social distancing and the breakdown of social networks, we should consider two major social theories — the social capital theory (SCT) and the social network theory (SNT). The social capital theory (SCT) was first defined by Bourdieu (1986) [21]. Social capital refers to the resources and benefits that individuals derive from their social networks and connections, which are made possible by accessing the social resources embedded within the network [21,22]. Social distancing measures which were imposed due to the COVID-19 pandemic, by limiting face-to-face interactions and reducing opportunities for social engagement, can disrupt the accumulation and maintenance of social capital. This disruption can result in the breakdown of social networks [23].

As for the structure, dynamics, and effects of social relationships, these are studied within another social theory — the social network theory (SNT) [24]. This theory investigates the influence of the social structure surrounding individuals, groups, or organizations on their beliefs and behaviors. Within the SNT context, social distancing measures can disrupt the structure of social networks by reducing the frequency and intensity of social interactions. Over time, the lack of regular contact and reduced opportunities for building and maintaining relationships due to pandemic restrictions may lead to shrinkage and breakdown of social networks [25]. Inevitably, this destroys social networks — as they have to be constantly produced and reproduced by interactions [[26].

In health research, the production and reproduction of social networks is analyzed from the viewpoint of their impact on people's behavior and health [27]. Loneliness, in particular, has been found to correlate with the quantity and especially the quality of social networks, where contacts with friends and neighbors have stronger associations with loneliness than contacts with family members. Other factors that correlate with loneliness are gender and age [28]. Aging has been shown to be associated with a reduction of social networks [29], which, in its turn, tends to increase perceived loneliness. A higher frequency of loneliness has been explained and predicted through the proximal and distal aspects of social networks [30]. Other studies confirm that older individuals with smaller social networks are more prone to feelings of loneliness than those with more social connections [31]. This has been proven to hold for health emergencies such as the COVID-19 pandemic, during which frequent face-to-face interactions with friends and family were associated with smaller increases in loneliness [32]. At the same time, the lack of social relationships experienced by older adults has been associated with loneliness during the pandemic [8]. Therefore, avoiding social contact in favor of limiting the spread of COVID-19 increased anxiety and stress in the population [33].

Additionally, there is accumulating evidence on the importance of social networks for health status. It was found that higher levels of social integration, such as more frequent contact with friends, family, neighbors, etc., have been associated with better physical health and fewer morbidities such as hypertension [34], heart disease [14], diabetes [35] and cancer [36]. Social networks were associated with lower overall mortality as they contributed to a reduction in deaths from cardiovascular disease, and strong social networks were linked to a lower incidence of stroke, and potential benefits in extending the survival rates of men with coronary heart disease [37]. Older adults were the most at-risk population for severe illness, hospitalization, intensive care unit admission, and death due to the pandemic [6]. They are subjective to loneliness and considered to be more socially isolated [38]. Therefore, the decrease in social contact due to the pandemic and social distancing policies had a major effect on the older population's health.

Due to social distancing restrictions, face-to-face interactions decreased, and the use of digital technologies surged [39]. Such digital communication contributed to alleviating the negative psychological consequences of the lockdown by decreasing the feeling of loneliness, anger, and boredom and fostering a greater sense of belongingness [40].

Since lack of social contact can affect physical health, changes in health status as a result of decreased social contact during the outbreak can be measured via a person's subjective self-health perception. Self-perceived health is a summary statement that contains a combination of several subjective and objective aspects of health according to the respondent's perception [41]. It is evaluated with a single item that rates an individual's overall health on a scale that generally ranges from excellent to poor [42]. Self-perceived health is a powerful indicator of the health status of older people [43] and is considered a valid and reliable indicator of overall health status [[44], [45], [46]] and a strong predictor of mortality [47]. Previous studies have found correlations between self-assessed poor health and chronic conditions, such as hypertension, heart disease, diabetes, cancer, stroke, and paralysis, among older people [[48], [49], [50], [51], [52]]. Although previous studies have examined the connection between social contact during the COVID-19 outbreak and mental health, little is known about the connection between social contact during the early COVID-19 pandemic and physical health.

### 1.1 Purposes

The objectives of this paper were to examine potential connections between:

• self-perceived changes in loneliness and self-perceived changes in health status stratified by gender within age groups,
• the existence and type of social contact (face-to-face/electronic) and self-perceived changes in health status stratified by gender within age groups,
• illnesses/health conditions and self-perceived changes in health status stratified by gender.^1^

For the purposes above, the study focused on adults aged 50 and older during the COVID-19 outbreak (June–August 2020).

## 2 Methods

### 2.1 Data source: SHARE survey

The COVID-19 data used in the study was obtained from the Survey of Health, Ageing and Retirement in Europe (SHARE) Wave 8 Release 0.0.1 beta [53], which is the first published SHARE dataset related to COVID-19. This data was collected between June and August 2020, during the initial stages of the COVID-19 outbreak, for 25 European Union countries (excluding Austria and Ireland), Israel and Switzerland.

SHARE is a longitudinal, cross-country database of health-related, economic, and social data. It provides data for about 140,000 individuals aged 50 or older (around 530,000 interviews) and was founded in 2002 [[54], [55], [56]]. This study analyzes the data provided by SHARE Wave 8 beta on self-perceived changes in health and loneliness, social contact type (face-to-face/electronic), and health conditions.

### 2.2 Sample characteristics and sampling process

A total of 51,778 individuals aged 50 and older (average age 70.5 ± 9.25 standard deviations) who responded to the changes in health question were stratified by gender within four age groups: 50–59, 60–69, 70–79, and 80+ (Table 1).

**Table 1:** Basic demographics of participants: age and gender.

Characteristic	N (% gender; % age group)		
Age group	**Male**	**Female**	**Both genders**
50–59	2,117 (34.9)	3,955 (65.1)	6,072 (11.7)
60–69	8,288 (43.5)	10,758 (56.5)	19,046 (36.8)
70–79	7,600 (44.4)	9,499 (55.6)	17,099 (33.0)
80+	3,873 (40.5)	5,688 (59.5)	9,561 (18.5)
Total	21,878 (42.3)	29,900 (57.7)	51,778 (100.0)

These individuals were analyzed for their changes in health and loneliness since the outbreak, as well as for the existence and type of social networks (face-to-face or electronic contact) and various health conditions (Table 2).

**Table 2:** Characteristics of participants and distribution of study parameters.

Characteristic	N (%)
Illness or health conditions:	
Diabetes or high blood sugar	1,021 (10.7)
High blood pressure or hypertension	2,350 (24.7)
Heart attack or other heart problem	1,405 (14.7)
Chronic lung disease	611 (6.4)
Cancer or malignant tumor	780 (8.2)
Other illness or health condition	3,365 (35.3)
Change in your health since the outbreak:	
Improved	1,489 (2.9)
Worsened	4,636 (9.0)
About the same	45,653 (88.2)
More or less lonely since outbreak:	
More so	5,985 (11.6)
Less so	447 (0.9)
About the same (and hardly ever or never lonely)	45,025 (87.5)
Social contact type:	
Face-to-face:	
Without	4,732 (17.5)
With (any)	22,299 (82.5)
Face-to-face contact since outbreak (any) with:	
Own children	38,482 (35.3)
Own parents	6,361 (5.8)
Other relatives	27,012 (24.8)
Neighbors, friends, or colleagues	34,918 (32.0)
Volunteered	2,333 (2.1)
Electronic:	
Without	1,313 (3.3)
With (any)	38,569 (96.7)
Electronic contact since outbreak (any) with:	
Own children	44,041 (31.5)
Own parents	7,573 (5.4)
Other relatives	44,380 (31.8)
Neighbors, friends, or colleagues	43,641 (31.3)
Face-to-face vs. Electronic:	
With face-to-face (any)	22,299 (36.6)
With electronic (any)	38,569 (63.4)

### 2.3 Self-perceived changes in health status since the outbreak

In the SHARE questionnaire, a question regarding self-perception of change in health condition was asked: “*If you compare your health with that before the outbreak of Corona, would you say your health has improved, worsened, or stayed about the same?*” with the options: “*Improved*”, “*Worsened*” or “*About the same*”. In this study, an individual's health was considered worsened when replied “*Worsened*” (n = 4,636) and considered not worsened when replied “*Improved*” or “*About the same*” (n = 47,142).

### 2.4 Self-perceived changes in loneliness since the outbreak

Two questions were asked regarding loneliness. The first was about the duration of feeling lonely: “*How much of the time do you feel lonely?*”. Individuals were offered three response options: “*Often*”, “*Some of the time*” or “*Hardly ever or never”*. The second question was about changes in loneliness since the outbreak: “*Has that been more so, less so or about the same as before the outbreak of Corona?*”. A total of 36,653 Individuals (71% of the sample) answered “*Hardly ever or never*” to the first question; therefore, the second question was not applicable for them to answer. Since these individuals “*Hardly ever or never*” feel lonely, this feeling did not change during the outbreak of Corona. Therefore, they were considered as feeling “*About the same*” since the outbreak. Loneliness since the outbreak was considered not increased if individuals replied “*Less so*” or “*About the same*” to the second question (including if the first question was answered “*Hardly ever or never*”, n = 45,472). Changes in loneliness since the outbreak were considered increased if an individual responded “*More so*” to the second question (n = 5,985).

### 2.5 Face-to-face social contact since the outbreak

Participants were asked questions regarding their face-to-face social contact since the outbreak i.e. “*Since the outbreak of Corona, how often did you have personal contact, that is, face-to-face, with the following people from outside your home? Was it daily, several times a week, about once a week, less often, or never?*”. The relationships were “*Own children*”, “*Own parents*”, “*Other relatives*” and “*Other non-relatives like neighbors, friends, or colleagues*”. An additional question was, “*Since the outbreak of Corona, did you do any volunteering activity*?” with “*Yes*” or “*No*” as an answer. Individuals were considered as having face-to-face social contact when they had any contact (volunteering was not considered and “*Not applicable*” was considered since there are older individuals without living parents, for example. Individuals that were “*Not applicable*” for all relationships were subtracted from the sample) (n = 22,299). Individuals were considered without face-to-face social contact if they replied “*Never*” or were “*Not applicable*” for face-to-face contact and replied “*No*” for volunteering (n = 4,732).

### 2.6 Electronic social contact since the outbreak

A question was asked regarding electronic social contacts since the outbreak: *“Since the outbreak of Corona, how often did you have contact by phone, email, or any other electronic means with the following people from outside your home? (Was it daily, several times a week, about once a week, less often, or never?)”* The relationships were “*Own children*”, “*Own parents*”, “*Other relatives*” and “*Other non-relatives like neighbors, friends, or colleagues*”. Individuals were considered as having electronic social contact when they had any contact (“*Not applicabl*e” was considered since there are older individuals without living parents, for example). Individuals who were “*Not applicable*” for all relationships were subtracted from the sample (n = 38,569). Individuals were considered without electronic social contact when they replied “*Never*” or were “*Not applicable*” for electronic contact (n = 1,313). Individuals with face-to-face social contact might also have electronic social contact and vice versa.

### 2.7 Illnesses or health conditions

Questions regarding the participant's illnesses or health conditions were asked: *“Do you have any of the following illnesses or health conditions? Please answer yes or no: With this we mean that a doctor has told you that you have this condition, and that you are either currently being treated for or bothered by this condition”* with the following options: *“Diabetes or high blood sugar?”* (n_(yes)_ = 1,021, n_(no)_ = 4,556), “*High blood pressure or hypertension?*” (n_(yes)_ = 2,350, n_(no)_ = 3,221), “*A heart attack including myocardial infarction or coronary thrombosis or any other heart problem including congestive heart failure?*” (n_(yes)_ = 1,405, n_(no)_ = 4,168), “*Chronic lung disease such as chronic bronchitis or emphysema?*” (n_(yes)_ = 611, n_(no)_ = 4,963), “*Cancer or malignant tumor, including leukemia or lymphoma, but excluding minor skin cancers?*” (n_(yes)_ = 780, n_(no)_ = 4,781) and “*Another illness or health condition*” (n_(yes)_ = 3,365, n_(no)_ = 2,218). Individuals might have more than one illness or health condition.

### 2.8 Statistical analysis

The analysis was carried out using SPSS, version 25.0 (IBM Corp., Armonk, NY, USA). Pearson chi-square (*χ*^2^(degrees of freedom)) and odds ratio (OR) (with a 95% confidence interval (95% CI)) statistical methods were utilized to examine the dependence and connection between self-perceived changes in loneliness, face-to-face/electronic contact, various health conditions and self-perceived changes in health status since the COVID-19 outbreak. The same statistical methods were used to investigate the effects of age and gender on the associations above. In cases where the expected count was less than 5, Fisher's exact test was performed (instead of Pearson chi-square). *p* values < 0.05 were considered significant.

In addition, it was decided to select household financial resilience since the outbreak as a control variable for analyzing the connection between self-perceived changes in loneliness and self-perceived changes in health status, and between social contacts and self-perceived changes in health status (subsection 1.5. in the Supplementary data: SHARE question on household financial resilience). For this, we conducted a hierarchical binary logistic regression analysis (section 2. in the Supplementary data: Analysis of results). However, due to non-significant differences in the results between the analysis with and without the control variable (95% CI highly overlapped), the Results section below will only refer to the analysis conducted without the control variable.

## 3 Results

### 3.1 Dependence and connection between self-perceived changes in loneliness and self-perceived changes in health status since the outbreak

**Fig. 1:**
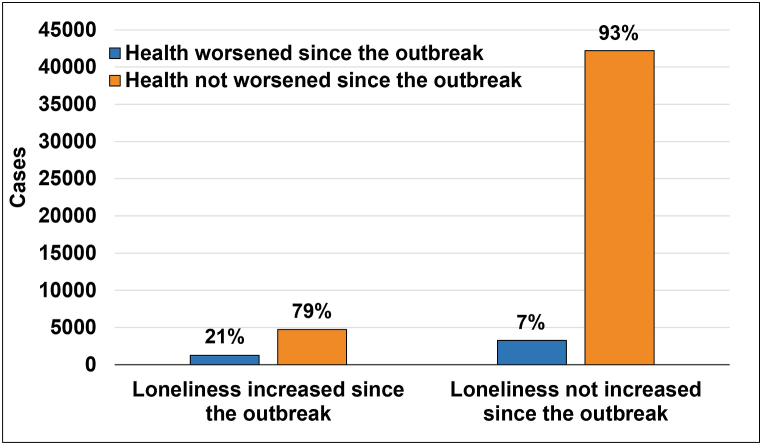
Self-perceived changes in health status as a function of self-perceived changes in loneliness since the outbreak.

To examine the connection between self-perceived changes in loneliness and health status since the outbreak, we evaluated individuals who reported an increase or no change in loneliness for their self-perceived health status during the same period (Fig. 1).

Chi-square and odds ratio analyses revealed a significant dependence and connection between self-perceived change in loneliness and health since the outbreak. The odds of health being worse since the outbreak were 249% higher (95% CI = 3.25–3.75) among individuals who experienced increased loneliness compared to those who did not since the outbreak (*χ*^2^(1) = 1,307.86, *p* < 0.001).

Stratification by gender within age groups demonstrated a significant dependence and connection across all comparisons (OR ranged from 2.9, 95% CI = 2.28–3.57, *p* < 0.001 to 4.6, 95% CI = 3.61–5.90, *p* < 0.001; Chi-square ranged from *χ*^2^(1) = 45.51, *p* < 0.001 to *χ*^2^(1) = 220.97, *p* < 0.001).

### 3.2 Dependence and connection between social contact and self-perceived changes in health status since the outbreak

**Fig. 2:**
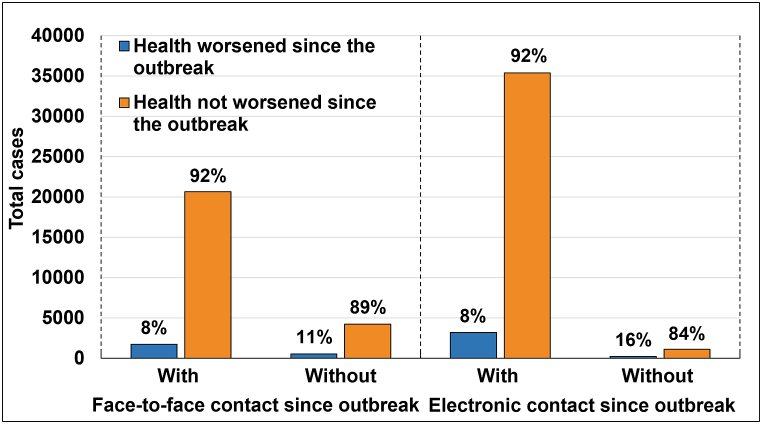
Self-perceived changes in health status as a function of social contact since the outbreak.

We examined the contribution of social contact to self-perceived changes in health status since the outbreak, separately for face-to-face and electronic contact. For this purpose, we evaluated individuals with and without each type of social contact for their changes in self-perceived health status since the outbreak (Fig. 2).

Chi-square and odds ratio analyses revealed a significant dependence and connection between face-to-face or electronic social contact and self-perceived changes in health status since the outbreak. The odds for self-perceived health to be worse since the outbreak were 31% lower (95% CI = 0.62–0.76) for individuals who had face-to-face contact (χ^2^(1) = 50.23, *p* < 0.001), and 54% lower (95% CI = 0.40–0.54) for individuals who had electronic contact (χ^2^(1) = 104.15, *p* < 0.001) compared to individuals who did not have face-to-face or electronic contact since the outbreak.

Stratification by gender within age groups for face-to-face social contact demonstrated a significant dependence and connection for the age group 50–59, only for females (OR = 0.45, χ^2^(1) = 12.96, *p* < 0.001). For the age group 60–69, significance was found for both females (OR = 0.75, χ^2^(1) = 4.67, *p* = 0.031) and males (OR = 0.62, χ^2^(1) = 8.32, *p* = 0.004). As for the age groups 70–79 and 80+, significance was observed only for males (OR = 0.68, χ^2^(1) = 8.08, *p* = 0.005, and OR = 0.61, χ^2^(1) = 10.80, *p* = 0.001, respectively). 95% CI overlapped between genders within age groups and ranged between 0.28–0.97.

For electronic social contact, significant dependence and connection were found for the age group 70–79, only for males (OR = 0.44, χ^2^(1) = 21.14, *p* < 0.001), and for the age group 80+ for both males (OR = 0.40, χ^2^(1) = 40.42, *p* < 0.001) and females (OR = 0.71, χ^2^(1) = 5.14, *p* = 0.024). 95% CI overlapped between genders within age groups and ranged between 0.30–0.96.

### 3.3 Dependence and connection between health conditions and self-perceived changes in health status since the outbreak

**Fig. 3:**
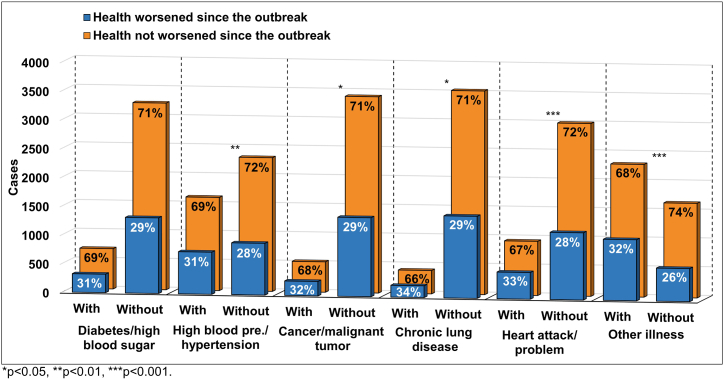
Self-perceived changes in health status as a function of health conditions.

To assess which health conditions or illnesses individuals reported worse self-perceived health status since the outbreak, we examined individuals with and without such conditions for their reported changes in health status since the outbreak (Fig. 3).

Chi-square and odds ratio analyses revealed a significant dependence and connection between health conditions and self-perceived changes in health status since the outbreak. The odds for health to be worse since the outbreak were 17%, 19%, 25%, 27%, and 32% (95% CI overlapped and ranged between 1.01–1.50) higher in individuals who reported high blood pressure or hypertension (χ^2^(1) = 6.75), cancer or malignant tumor (χ^2^(1) = 4.18), chronic lung disease (χ^2^(1) = 6.10), heart attack and other heart problem (χ^2^(1) = 12.70) and other illness or health condition (χ^2^(1) = 20.49), respectively.

Stratification by gender demonstrated that worse self-perceived health had a significant dependence on and connection with high blood pressure or hypertension in females (OR = 1.17, χ^2^(1) = 3.93, *p* = 0.048). Another health condition that correlated with worse self-perceived health in females was diabetes or high blood sugar (OR = 1.22, χ^2^(1) = 4.13, *p* = 0.042). For males, worse self-perceived health had a significant dependence on and connection with cancer or malignant tumor (OR = 1.42, χ^2^(1) = 8.29, *p* = 0.004), and chronic lung disease (OR = 1.47, χ^2^(1) = 8.34, *p* = 0.004). As for heart attack or other heart problems as well as other illnesses or health conditions, they showed a significant dependence on and connection with worse self-perceived health in both males and females (OR = 1.27, χ^2^(1) = 6.27, *p* = 0.012, and OR = 1.28, χ^2^(1) = 7.08, *p* = 0.008 for heart attack or other heart problems, males and females, respectively; OR = 1.31, χ^2^(1) = 9.04, *p* = 0.003, and OR = 1.32, χ^2^(1) = 11.07, *p* = 0.003 for other illnesses or health conditions, males and females, respectively). 95% CI overlapped between genders and ranged between 1.00–1.91.

## 4 Discussion

This study examined the connection between self-perceived changes in loneliness and the existence and type of social contact (face-to-face or electronic) on self-perceived changes in health status by gender within age groups in adults aged 50 plus during the COVID-19 outbreak. Additionally, the study examined which health conditions were related to worsened self-perceived health status.

We found that the odds for worsened self-perceived health status were higher among individuals with increased self-perceived loneliness compared to the non-increased self-perceived loneliness group during the outbreak (controlling for household financial resilience showed no significant effect) beyond age and gender. This finding broadly supports the work of other studies in this area linking self-perceived loneliness and health status among older persons [[57], [58], [59]].

In the current study, face-to-face contact served as a protective factor for worsened self-perceived health status (controlling for household financial resilience showed no significant effect). Our study supports evidence from other observations that measured social networks in three areas: friends, extended family, and children, quantified by combining the monthly frequency of face-to-face and telephone contact, intimacy, and proximity [60]. The study showed that social networks demonstrated unique positive associations with health among older people, although it does not appear to be affected by gender [60]. We included volunteering as face-to-face contact since it is known that there is a strength of association between social participation and positive self-perceived health [61]. The relevance of face-to-face contact's contribution to health status changed gradually between genders among age groups. First, it served as a protective factor among relatively younger women (aged 50–59). Second, in both genders (aged 60–69), and only in older males (aged 70 plus). Therefore, maintaining high face-to-face contact among younger females and older males could lower their worsening self-perceived health status. In accordance with the present results, previous studies have demonstrated that the association between social interactions and self-rated health varies between genders and that low social network involvement was associated with poor self-rated health in older men [18]. In addition, men aged 50 plus tend to maintain intimate relationships with only a few people, while women identify more people as being important to them or as people they care about [62].

In general, electronic communication was found to be more beneficial for self-perceived health than face-to-face communication (controlling for household financial resilience showed no significant effect), and was only observed in males aged 70–79 and in both genders aged 80 plus. For this, we can offer several explanations. First, while recognizing that physical contacts are still considered very important, we rely on evidence suggesting an increasing tendency for individuals to communicate via electronic channels. One such research conducted in 2010 proves that the choice of the communication channel depends on the content and urgency of the message to be communicated [63]. Based on the survey of 742 participants, the study concludes that irrespective of the type of contact (ranging from relatives to colleagues), urgent matters are communicated predominantly via the electronic mode (from 70.6% to 91.7%, respectively), being represented almost exclusively by phone calls (from 67.5% to 72.8%, respectively). For important but not urgent messages, the electronic mode remains preferable in communication (from 76.5% for relatives to 89.2% for colleagues). Here, phone calls still show the highest frequency for relatives and close friends (60.6% and 54.2% respectively) [63]. We would like to stress that the study above, being conducted in February–August 2007, was not affected by the state of emergency related to COVID-19. During the COVID-19 pandemic, messages obviously became more urgent and important — as individuals found themselves more concerned about their own health, the health of the people they knew, and the general situation in the world. In this light, the results obtained in our study (of a higher connection of perceived health status with electronic rather than face-to-face communication) seem rooted in the type of content communicated in the early-Corona wave.

Another possible explanation of this (seemingly) paradoxical finding may be ‘collective anxiety’ and ‘a global epidemic of anxiety’ experienced in Europe during the COVID-19 pandemic [64]. One of the biggest factors inducing anxiety in the population of whole states and continents during COVID-19 was the intense fear of catching SARS-CoV-2. To this, we can add the influence of massively circulated fake news and conspiracy theories flooding all media, particularly during the onset of the pandemic [64]. As a result of this intense fear of catching the disease, people preferred electronic communication that was risk-free — quite in line with our findings.

Although the connection between electronic contact and self-perceived health status has not been established yet in the literature, the beneficial effect of electronic communication on mental health is inconclusive. One study suggests that electronic contacts significantly increased negative mental health changes measured during the COVID-19 outbreak [65], while another research shows that online social participation buffered the effects of pain on mental well-being [66]. These studies differ in database use, data handling, and statistical approaches. In contrast to the research above that focuses on mental health, this study examines the contribution of electronic communication to self-perceived health status, which was found to be more beneficial than the contribution of face-to-face communication.

Previous studies found correlations between self-assessed poor health and the number of chronic diseases among older people [[48], [49], [50], [51], [52]]. This study screened several health conditions for their connection to changes in self-perceived health status since the outbreak. We found that hypertension was connected to worsened self-perceived health status. It has been suggested that individuals with hypertension and high blood pressure may be at increased risk from the effects of COVID-19 [67,68]. Based on this, it is important for older individuals with hypertension to follow social distancing rules. In addition, Laffin et al. [69] referred to a rise in blood pressure levels during the pandemic. Possible reasons for this included higher alcohol consumption, reduced physical activity, emotional stress, and limited access to ongoing medical care, leading to reduced medication adherence. In our study, stratification by gender revealed a significant connection between hypertension or high blood pressure and worse self-perceived health status only for females. Similarly, the study above [69] found that women exhibited higher increases in blood pressure than men between April–December 2020 versus 2019. This applied to all age groups under study, including adults aged 50 to 88. Their explanation for this is rooted in the overall impact of pandemics on the female population, implying that the social and economic effects of outbreaks tend to disproportionately affect women compared to men.

For diabetes, this study did not find a connection with worsened self-perceived health when analyzed without stratification by gender. Similarly, according to a study conducted in Belgium, there was no increase in hospitalization for COVID-19 in subjects with type 1 diabetes at the onset of the pandemic (February–April 2020) [70]. However, stratification by gender revealed a significant connection between diabetes or high blood glucose levels and worse self-perceived health status, but only for females. Priya et al. [71], in their review of the challenges in women with diabetes during the pandemic, suggested that women are a vulnerable group. This may be due to a higher burden of comorbidities and micro- and macro-vascular complications compared to men.

Cancer was also connected to worsened self-perceived health status since the outbreak when analyzed without stratification by gender. One study found that COVID-19 patients with cancer had mortality odds of 134% in severe outcomes and that patients with stage four hematological cancers, lung cancer, or metastatic cancer had the highest frequency of major adverse events [72]. Hence, it was crucial for older individuals with cancer to adhere to the recommended social distancing guidelines. In this study, when analyzing the data by gender, we found a significant connection between cancer and worse self-perceived health, but only among males which is described below.

We found that individuals who reported having chronic lung disease had relatively increased worse self-perceived health status. Since the COVID-19 virus is caused by SARS-CoV-2, it can affect the upper and lower respiratory tract [73]. Therefore, individuals with chronic lung disease are more likely to become severely ill when they have COVID-19 complications [74], which can temporarily worsen respiratory symptoms [75]. One explanation for the increased worse self-perceived health status in this group could be the combined risk of older age and respiratory disease, which makes this group most vulnerable to the COVID-19 pandemic, as the lungs are a primary target for the coronavirus. These vulnerable patients should carefully follow the guidelines to minimize their risk of infections that could cause inflammation and breathing problems by staying home and physically distancing themselves from others. However, this results in less outside physical activity, which decreases the ability to improve health and reduce stress. Since self-perceived health status has physical and mental components [60,76], this group reported worse self-perceived health status. Stratifying the data by gender revealed a significant connection between chronic lung disease and worse self-perceived health status, but only for males. In this context, a study conducted between 1993 and 2003 on European adults aged 55 to 74 found that the prevalence of current cigarette (Tobacco) smokers was higher among men than women when the difference in smokers prevalence ranged between 10–15% [77]. Another study conducted during the pandemic found that among individuals who exclusively smoked, there was an observed increase in their smoking frequency. This tendency appeared to be influenced by factors such as COVID-19-related stress, extended periods spent at home, and feelings of boredom. This particular group was not driven to quit smoking during the pandemic, and a subset of them believed that smoking could offer some level of protection against COVID-19 [78]. Among various risk factors for chronic obstructive pulmonary disease (COPD), tobacco smoke stands out as the primary contributor [79], constituting more than 70% of COPD cases in high-income countries [80]. In addition, tobacco use has the potential to trigger cancer in various parts of the body, such as the lungs, head and neck, bladder, kidney, liver, stomach, pancreas, colon, and rectum. It is worth noting that nearly nine out of every ten cases of lung cancer are directly caused by smoking [81]. Given the higher prevalence of smoking among men compared to women before the pandemic, coupled with the circumstances of the pandemic leading to an increase in smoking frequency for both genders, it is possible that this could have contributed to continued smoking habits and worsened health outcomes for individuals, particularly men.

In addition, we found that individuals who reported suffering from heart attack and other heart problems had increased worsened self-perceived health status. It is known that stress and heart conditions are positively related [82]. As a result of the COVID-19 outbreak, population stress levels increased drastically due to several stressors, such as financial concerns (reported as the most stressful factor), media reports generating virus-related anxiety, uncertainty about the future, and disruptions in established routines [33]. In addition, Bromage et al. [83] reported a significantly lower rate of hospitalizations for acute heart failure during the COVID-19 pandemic and more severe symptoms at the time of hospital admission. One hypothesis for this pattern suggests that patients might have refrained from seeking medical attention at hospitals during the national lockdown and social distancing restrictions [83]. Therefore, many individuals reported that their self-perceived health status worsened, which manifested in heart problems during the outbreak. This indicates that the policy of social alienation imposed on older individuals was linked to adverse changes in their self-perceived health.

### 4.1 Limitations

This study had some limitations. One, the survey is based on self-report questionnaires and a person's subjective experience of self-health perception. Since the SHARE database does not contain medical records, we do not know whether an individual's health status actually worsened. However, we found a significant connection and dependence between the worsening of self-perceived health status and several health conditions. In addition, a person's subjective feelings about mental and medical conditions are representative because self-perceived health is considered a reliable and valid indicator of the overall health status of older people [[44], [45], [46]]. Another limitation is that the study sample included more females than males, which might result in unrepresentative results. A high woman-to-man sample size ratio also was found in several studies since females tend to participate in surveys more frequently than males [84,85]. However, the connection between self-perceived health changes and loneliness, and self-perceived health changes and social networks analyses were conducted separately for both genders within age groups, which found no differences in gender ratios compared to the total sample.

Furthermore, while our findings suggest that electronic communication may serve as a buffer against the adverse effects of pandemic-induced social restrictions, it is important to note that the specific purpose or nature of these electronic contacts was not investigated in this study due to limited information available in the SHARE database. Future studies could explore the various forms and dynamics of electronic communication to gain a more comprehensive understanding of its role in mitigating the adverse effects of social restrictions during the pandemic.

We view another limitation of the current study in a certain inconsistency of terms inherited from the SHARE database. While referring to gender, SHARE distinguishes between males and females — instead of men and women. We recognize that gender identity is constructed socially (not biologically) and is not confined to a binary opposition [86]. The database should therefore have referred to sex rather than gender. At the same time, it seemed important to align our analysis with the original wording used in the SHARE database and to be consistent with how this variable is categorized in the SHARE database.

## 5 Conclusions

The pandemic has created a positive incentive (catalyst) for the use of electronic communication due to social distancing restrictions. This shift was especially essential for older individuals with chronic diseases. Based on the results obtained, electronic contact could serve as a protective factor and may help to buffer or ease the aggravation of the perception of health among older individuals. In addition, our study found that both increased self-perceived loneliness and several health conditions, such as heart and lung diseases, were connected to worsened self-perceived health. Therefore, adopting a policy that encourages the use of electronic communications could relieve health authorities of the burden on health services while improving patient health outcomes and minimizing health risks. This seems especially important during pandemics and for older people, as any departure from home can cause an additional risk of exposure to the virus. It is important to note that electronic communication requires internet connection and technology that are less available to the older population, individuals with chronic illness, and individuals living in poverty. Therefore, policymakers may consider implementing strategies that will increase the availability of electronic communication by offering affordable access to relevant communication technologies.

Due to the potential uncertainty of additional viruses emerging in upcoming years, and the inability to prepare in advance with proactive vaccination, the population should be able to cope with quarantine and social limitations. Therefore, it is very important to nurture the use of social networks among the population, especially for older individuals with health conditions and chronic illnesses. This, in turn, could positively influence their mental and physical health, help save money in the long term and relieve health authorities of the burden on health services. Additional research should be conducted to evaluate loneliness and social networks against objective medical records collected during the COVID-19 outbreak.

## Author contribution statement

Shay Musbat: Conceived and designed the experiments; Performed the experiments; Analyzed and interpreted the data; Contributed reagents, materials, analysis tools or data; Wrote the paper.

Inbal Reuveni: Analyzed and interpreted the data.

Racheli Magnezi: Conceived and designed the experiments; Analyzed and interpreted the data; Wrote the paper.

## Data availability statement

SHARE data is free of charge for scientific use globally (available at http://www.share-project.org/data-access.html).

## Ethical standards statement

Ethical approval was not required for this study since it was based solely on secondary data analysis (see www.share-project.org).

## *Participant* consent *statement*

Participant consent was not required for this study since it was based solely on secondary data analysis (see www.share-project.org).

## Funding *statement*

This research did not receive any specific grant from funding agencies in the public, commercial, or not-for-profit sectors.

## Declaration of competing interest

The authors declare that they have no known competing financial interests or personal relationships that could have appeared to influence the work reported in this paper.

## Footnotes

1 Without stratification within age groups, to keep the representativeness of each sample.
