# Supplementary material for: Social networks as a protective factor for worsened self-perceived health status related to self-perceived changes in loneliness and health conditions in adults aged 50+ during the COVID-19 outbreak
Source: Heliyon. 2023 Sep 29;9(10):e20529. doi: 10.1016/j.heliyon.2023.e20529 (PMC10582293; doi:10.1016/j.heliyon.2023.e20529)
Supplement: Multimedia component 1 [file mmc1.docx]

**Supplementary data**

**Social networks as a protective factor for worsened self-perceived health status related to self-perceived changes in loneliness and health conditions in adults aged 50+ during the COVID-19 outbreak**

**Shay Musbat ^a^, Inbal Reuveni ^b^, Racheli Magnezi ^a,*^**

^a^ Department of Management, Health Systems Management Program, Bar-Ilan University, Ramat Gan 5290002, Israel

^b^ Department of Psychiatry, Hadassah Hebrew University Medical Center, Ein Kerem, Jerusalem 9112001, Israel

*** Corresponding author.** Email address: [Racheli.Magnezi@biu.ac.il](mailto:Racheli.Magnezi@biu.ac.il).

**1. SHARE questions used for the paper**

The questions used for the paper were taken from the SHARE COVID-19 questionnaire for telephone interviews (June 2nd, 2020). SHARE COVID-19 questionnaire for telephone interviews is available online: <https://share-eric.eu/fileadmin/user_upload/Questionnaires/Corona_Questionnaire_1/corona_en_cati-Generic.pdf>. The letters and numbers combination in the parentheses is the question code used by the SHARE COVID-19 questionnaire.

**1.1. Self-perceived changes in health status since the outbreak question:**

HEALTH: CHANGE IN YOUR HEALTH SINCE THE OUTBREAK (CAH002_)

Question

If you compare your health with that before the outbreak of Corona, would you say your health has improved, worsened, or stayed about the same?

Response options:

1. Improved

2. Worsened

3. About the same

**1.2. Self-perceived changes in loneliness since the outbreak questions:**

HEALTH: HOW OFTEN DO YOU FEEL LONELY (CAMH037_)

Question 1

How much of the time do you feel lonely? Often, some of the time, or hardly ever or never?

Response options:

1. Often

2. Some of the time

3. Hardly ever or never

HEALTH: MORE OR LESS LONELY SINCE OUTBREAK (CAMH837_)

Question 2

Has that been more so, less so or about the same as before the outbreak of Corona?

Response options:

1. More so

2. Less so

3. About the same

**1.3. Social networks questions:**

SOCIAL: CONTACT FREQUENCY - FACE-TO-FACE (CAS003_)

Question

Since the outbreak of Corona, how often did you have personal contact, that is, face to face, with the following people from outside your home? Was it daily, several times a week, about once a week, less often, or never?

*IWER: Read out each relationship and check the appropriate answer.*

Own children: (CAS003_1)

Own parents: (CAS003_2)

Other relatives: (CAS003_3)

Other non-relatives like neighbors, friends, or colleagues: (CAS003_4)

Response options:

1. Daily

2. Several times a week

3. About once a week

4. Less often

5. Never

99. Not applicable

-1. Don't know

-2. Refusal

SOCIAL: CONTACT FREQUENCY - ELECTRONIC (CAS004_)

Question

Since the outbreak of Corona, how often did you have contact by phone, email or any other electronic means with the following people from outside your home? (Was it daily, several times a week, about once a week, less often, or never?)

*IWER: Read out each relationship and check the appropriate answer.*

Own children: (CAS004_1)

Own parents: (CAS004_2)

Other relatives: (CAS004_3)

Other non-relatives like neighbors, friends, or colleagues: (CAS004_4)

Response options:

1. Daily

2. Several times a week

3. About once a week

4. Less often

5. Never

99. Not applicable

-1. Don't know

-2. Refusal

**1.4. Illnesses or health conditions questions:**

HEALTH (CAH004_)

Question

Do you have any of the following illnesses or health conditions? Please answer yes or no:

*IWER: With this we mean that a doctor has told you that you have this condition, and that you are either currently being treated for or bothered by this condition.*

*IWER: READ OUT.*

Hip fracture? (CAH004_1)

Diabetes or high blood sugar? (CAH004_2)

High blood pressure or hypertension? (CAH004_3)

A heart attack including myocardial infarction or coronary thrombosis or any other heart problem including congestive heart failure? (CAH004_4)

Chronic lung disease such as chronic bronchitis or emphysema? (CAH004_5)

Cancer or malignant tumor, including leukemia or lymphoma, but excluding minor skin cancers? (CAH004_6)

An other illness or health condition (CAH004_7)

Response options:

1. Yes

5. No

-1. Don't know

-2. Refusal

**1.5. Household financial resilience since the outbreak as a control for the association between self-perceived changes in loneliness or social contacts and self-perceived changes in health status question:**

ECONOMIC: HOUSEHOLD'S ABILITY TO MAKE ENDS MEET SINCE OUTBREAK (CACO007_)

Question

Thinking of your household's total monthly income since the outbreak of Corona, would you say that your household is able to make ends meet with great difficulty, with some difficulty, fairly easily, or easily?

Response options*:

1. With great difficulty

2. With some difficulty

3. Fairly easily

4. Easily

* The responses were analyzed dichotomously, where options 1 and 2 correspond to 'With difficulty', and options 3 and 4 correspond to 'Without difficulty'.

**2. Control for household financial resilience since the outbreak in examining the association between self-perceived changes in loneliness or social contacts and self-perceived changes in health status**

**2.1. Self-perceived changes in loneliness and self-perceived changes in health status (control: household financial resilience)**

| **Variables** | **B** | **S.E.** | **Odds ratio** | **95% C.I. for EXP(B)** | **Sig.** |
| --- | --- | --- | --- | --- | --- |
| **Constant** | 2.72 | 0.028 | 0.07 |  | |
| **Household financial resilience (With difficulty)** | 0.40 | 0.038 | 1.50 | 1.39-1.66 | *p* < 0.001 |
| **Loneliness change (increased)** | 1.21 | 0.043 | 3.36 | 3.09-3.65 | *p* < 0.001 |

In connection with worse self-perceived health. Based on 35,046 individuals who answered all the three questions.

**2.2. Face-to-face social contact and self-perceived changes in health status (control: household financial resilience)**

| **Variables** | **B** | **S.E.** | **Odds ratio** | **95% C.I. for Odds ratio** | **Sig.** |
| --- | --- | --- | --- | --- | --- |
| **Constant** | 2.72 | 0.028 | 0.07 |  | |
| **Household financial resilience (With difficulty)** | 0.46 | 0.054 | 1.59 | 1.43-1.76 | *p* < 0.001 |
| **Face-to-face contact (with)** | -0.30 | 0.067 | 0.74 | 0.65-0.84 | *p* < 0.001 |

In connection with worse self-perceived health. Based on 18,181 individuals who answered all the three questions.

**2.3. Electronic social contact and self-perceived changes in health status (control: household financial resilience)**

| **Variables** | **B** | **S.E.** | **Odds ratio** | **95% C.I. for Odds ratio** | **Sig.** |
| --- | --- | --- | --- | --- | --- |
| **Constant** | -2.10 | 0.122 | 0.12 |  | |
| **Household financial resilience (With difficulty)** | 0.49 | 0.043 | 1.63 | 1.50-1.77 | *p* < 0.001 |
| **Electronic contact (with)** | -0.47 | 0.122 | 0.62 | 0.49-0.79 | *p* < 0.001 |

In connection with worse self-perceived health. Based on 27,131 individuals who answered all the three questions.
